# A Novel Method for Measuring In-Shoe Navicular Drop during Gait

**DOI:** 10.3390/s120911697

**Published:** 2012-08-27

**Authors:** Simon L. Kappel, Michael S. Rathleff, Dan Hermann, Ole Simonsen, Henrik Karstoft, Peter Ahrendt

**Affiliations:** 1 Signal Processing and Control Group, Department of Engineering, Aarhus University, Finlandsgade 22, Aarhus N 8200, Denmark; E-Mails: hermann.dan@gmail.com (D.H.); hka@iha.dk (H.K.); pah@iha.dk (P.A.); 2 Orthopaedic Surgery Research Unit, Aalborg Hospital-Aarhus University Hospital, Denmark, Sdr. Skovvej 15, Aalborg 9000, Denmark; E-Mails: misr@rn.dk (M.S.R.); ohs@rn.dk (O.S.)

**Keywords:** capacitive strain sensor, gait analysis, foot movement, foot dynamics, Danfoss PolyPower, dielectric electro active polymer (DEAP), navicular drop, multi-video sequence analysis

## Abstract

Analysis of foot movement is essential in the treatment and prevention of foot-related disorders. Measuring the in-shoe foot movement during everyday activities, such as sports, has the potential to become an important diagnostic tool in clinical practice. The current paper describes the development of a thin, flexible and robust capacitive strain sensor for the in-shoe measurement of the navicular drop. The navicular drop is a well-recognized measure of foot movement. The position of the strain sensor on the foot was analyzed to determine the optimal points of attachment. The sensor was evaluated against a state-of-the-art video-based system that tracks reflective markers on the bare foot. Preliminary experimental results show that the developed strain sensor is able to measure navicular drop on the bare foot with an accuracy on par with the video-based system and with a high reproducibility. Temporal comparison of video-based, barefoot and in-shoe measurements indicate that the developed sensor measures the navicular drop accurately in shoes and can be used without any discomfort for the user.

## 1. Introduction

The human foot comprises a lightweight complex system combining passive and active elements which act in conjunction allowing for weight transfer, maintenance of balance and generation of propulsive forces during gait. Thus its function or malfunction will have effect on the rest of the body and *vice versa*. Measurement of foot function during activity is a requirement to provide meaningful interventions for treatment or prevention of foot-related disorders.

Several methods exist for measuring how the foot moves during bare foot walking. The two most commonly used methods include 3D motion capture using optical systems [1,2] or the use of inertial sensors [3,4]. Both methods require the foot to be visible to the cameras or require cutting a hole in the shoe or using sandals so that the inertial sensors can be attached directly to the foot. However, measurement of foot movement should be done in situations that reflect everyday life and normal loading of the foot. Therefore it is necessary to measure how the foot moves inside the shoe that people normally wear during everyday life. Measurement of in-shoe foot movement enables the study of how the foot and shoe interacts. A few attempts have been made to measure in-shoe foot movement but none of these has been successful in creating a method that is feasible in clinical practice. Current methods to measure in-shoe foot movement rely on specially designed shoes [5,6], radiostereometric analysis (RSA) [7,8] and dynamic MRI [9,10]. All these methods are expensive, time consuming, and not suitable for routine clinical use.

One of the methods currently used in clinical practice is measurement of the navicular drop [11]. The navicular drop describes the range of sagittal deformation of the midfoot during the stance phase of gait. Depending of the foot size the dynamic navicular drop for healthy persons is on average 5.3 mm (±1.8 mm) [12] but can vary up to 15 mm in problematic cases. The dynamic navicular drop is measured as the change in the navicular height (NH) from the time of heal strike (HS) to the time of lowest navicular height (NHL) during the stance phase of gait [13] The navicular drop has been suggested to be the most appropriate parameter for the assessment of foot pronation [14] as it is a valid indicator of talonavicular motion [8] and rear foot movement [15]. The size of navicular drop appears to have important consequences for subjects who participate in weight bearing sports such as running. Too much movement of the navicular (*i.e.*, the foot collapses during loading) places the subject in higher risk of developing injuries to the medial side of the shin [16,17] as well as the knee [18]. It is believed that when the navicular moves excessively and the foot collapses, it causes increased forces being transmitted to the tibia (shin bona) as well as increased internal rotation of the tibia which alters the biomechanics of the lower extremity [19,20].

In a previous project a Multi Video Sequence Analysis (MVSA) system was developed. The system is described in [13]. The MVSA system measures the navicular drop during gait, by using a camera to record the movement of a set of markers attached to the skin on the medial side of the foot. Research has shown that MVSA is a reliable technology to measure bare foot dynamic navicular drop with an accuracy of 0.5–0.8 mm [13]. However, the major shortcoming of the MVSA system is that it is limited to measuring the navicular drop during walking on a treadmill and only with bare feet.

Thus, the objective of this study was to develop a system that could meet the limitations of the MVSA system, by enabling a dynamic measurement of foot movement during in-shoe walking. The navicular drop was used to measure foot movement, and the MVSA system was used as reference system. The focus of the current study was to develop a thin flexible strain sensor that measures distance between the two endpoints, enabling a dynamic measurement of in-shoe navicular drop.

Strain sensors have been made with many different techniques such as capacitive strain gauges [21], conductive polymer composite [22–24] and dielectric electroactive polymer (DEAP) [25]. DEAP was chosen due to flexibility, thickness and accuracy of the measured strain of sensor.

## 2. Methods

In the following we describe a new type of strain sensor, data acquisition as well as the optimal position of a strain sensor on the foot.

### 2.1. The Newly Developed Strain Sensor

The new strain sensor is based on a DEAP material produced by Danfoss PolyPower. The material is called PolyPower and acts as an elastic capacitive material [25] that is strainable in one direction (Table 1).

**Figure 1.**
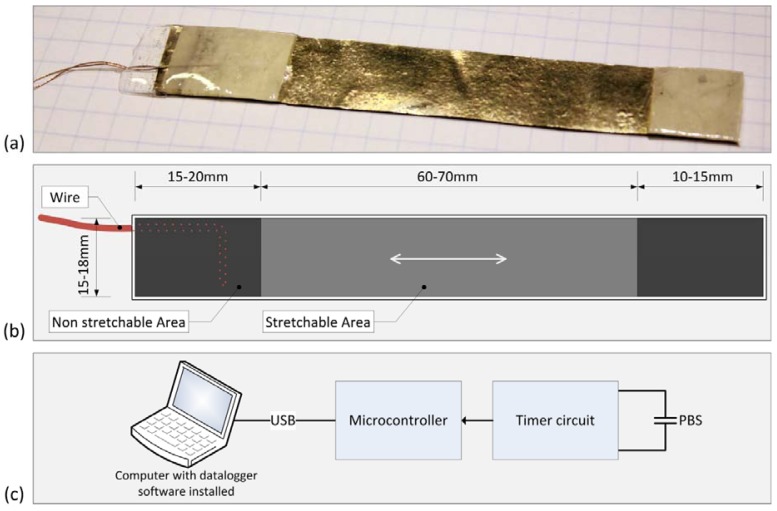
(**a**) The PBS, made from sheets of PolyPower; (**b**) Schematic drawing of the sensor, the non-strainable area is used to attach the sensor to the skin, and the strainable area measures the strain between two points of attachment; (**c**) Block diagram of the PBS system measuring the capacitance of the connected PBS.

Based on sheets of PolyPower, we designed a PolyPower based sensor (PBS) (Figure 1(a,b)). The sensor is mechanically stable, reusable, portable and resistant to perspiration from the foot. The sensor enables measurements of dynamic strain between two points of attachment on the medial side of the foot.

The non-strainable area of the developed PBS has a maximal thickness of 1.5 mm, and the strainable area has a thickness in the range of 0.40 mm–0.60 mm and can withstand a maximal strain of 50% of the strainable area. The thickness of a sensor enables it to be easily strainable, and comfortable to wear. For this reason a sensor has little impact on gait and foot movement. The thickness of a sensor also enables in-shoe measurements.

To measure the change in capacitance of a PBS, an electrical circuit was built with a timer (Figure 1(c)). The circuit was built in such a way that a change in capacitance of a PBS corresponded to a change in the timer frequency. The frequency from the timer circuit was measured by a microcontroller using a sample rate of 200 Hz which enable measurements of the navicular drop during running. The method enables measurements of the capacity with a resolution of 0.13 pF and a bandwidth of 200 Hz. To minimize the influence of parasitic electrical capacitances, the timer circuit was attached to the ankle of the test person to reduce the length of the wires from the sensor.

The data samples were transferred to a computer via a USB cable and were collected in a data logger program on the computer. The data logger program enabled real time visualization and logging of the data.

### 2.2. Estimation of the Optimal Position of a Strain Sensor to Measure the Navicular Drop

Measuring the navicular drop using a strain sensor is not straight forward, as the strain sensor is only capable of measuring the strain between two points of attachment on the foot. For this reason we performed an analysis to locate an estimate of the two optimal points of attachment. The analysis was based on measurements acquired using the MVSA system. The measurements were performed on the test person described in Table 2. Data from 20 consecutive steps were collected during walking on a treadmill. The person was asked to choose his preferred walking speed [26], which was 5 km/h.

**Figure 2.**
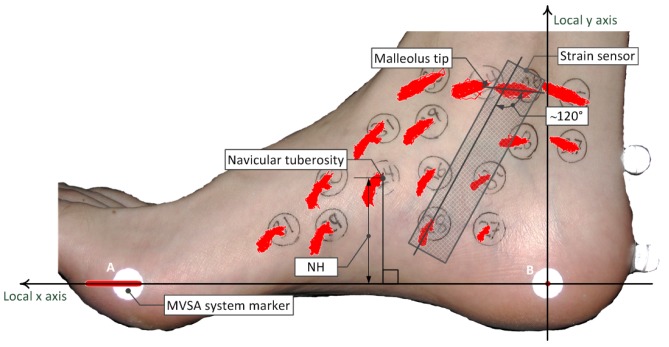
A set of measurements were performed using the MVSA system, and the measurements were combined, to locate the optimal position of a strain sensor. The red point clouds on different positions of the foot illustrate the marker displacement during gait at that particular position relative to the points A and B. The optimal position is illustrated by position of the strain sensor.

The cameras of the MVSA system have a frame rate of 114 Hz, and the pixel coordinates of the markers placed on the foot were calculated for each recorded picture. Two reference points on the foot were used to construct a local coordinate system that could be used to calculate the relative marker displacement. The marker placed at the medial side of the calcaneus (point B Figure 2) was set to have the local coordinate (0,0) in all pictures. The front foot marker (point A Figure 2) was set to have the local coordinate (x,0) in all pictures, where x is the local x coordinate (Figure 2). The local coordinate system was fitted on a picture of the foot, and the movement of the markers could be estimated as the change in the local coordinates from picture to picture. Movement of each marker during gait is illustrated as the red point clouds in Figure 2.

The measurements of the markers displacement illustrates that the movement of the navicular tuberosity corresponds to the movement of the medial side of the foot. For this reason the lower point of attachment does not have to be at the navicular tuberosity.

A very important requirement when we chose the optimal position of the sensor, was that the sensor should not be affected by pressure on the foot. For this reason the sensor must be in contact with the skin between the two points of attachment. To measure the movement of the navicular tuberosity, we chose a position where the movement of the upper point of attachment had the least impact on the strain of the sensor. This point was estimated to be where the major axis of the point cloud of the position close to the tip navicular was close to perpendicular to the major axis of the point cloud of the position close to the malleolus tip.

The optimal upper point of attachment was estimated to be approximately 20 mm behind the tip of the medial malleolus, and the optimal lower point of attachment was estimated to be approximately 20 mm behind and approximately 20 mm below the navicular tuberosity. When the sensor is located at this position, it is in contact with the skin between the two points of attachment. On the test persons foot the angle between the major axis of the point clouds of the points of attachment was found to be approximately 120° (Figure 2). The angle will most likely change from person to person, but the measurements indicate that the movement of the upper point of attachment will have a minimal impact on a strain sensor positioned at the given position.

### 2.3. Validation of the PBS System with the MVSA System

**Figure 3.**
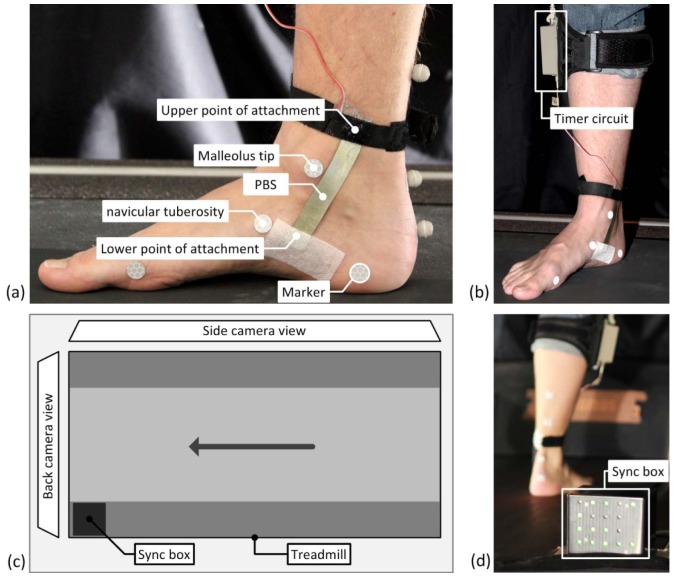
(**a**) The PBS and a number of markers were attached to the bare foot to acquire simultaneous measurements of the navicular drop; (**b**) The timer circuit was used to measure the current capacitance of the sensor. The timer circuit was attached to the ankle using a Velcro band; (**c**) The sync box was located in field of view of the back camera of the MVSA system; (**d**) The sync box contained LED's to display the measurement number that was recorded by the back camera of the MVSA and a microcontroller to measure the frequency from the timer circuit.

To be able to validate the PBS system with the MVSA system, the data from the two systems needed to be synchronized. A synchronization box (sync box) containing a microcontroller and 18 LEDs was created to enable synchronization of the samples from a PBS and the MVSA system during walking and running (Figure 3(d)). The sync box was positioned in the field of view of the back camera of the MVSA system (Figure 3(c)). The microcontroller measures the frequency of the timer circuit (Figure 3(b)) and was integrated in the sync box. Two of the LEDs on the sync box were left turned on, while the remaining 16 LEDs were used to display the measurement number in gray code [27]. Hence, the recordings from MVSA and PBS could be synchronized.

A Matlab^®^ script was used to recognize the PBS sample number displayed by the LEDs in the pictures from the MVSA system. This enabled sample by sample synchronization of the samples from MVSA system and the PBS system, respectively. Since the sample rate of the PBS system is 200 Hz and the frame rate of the MVSA system is 114 Hz, an interpolation algorithm was used to upsample the data from the MVSA system.

A synchronized navicular drop was calculated for each step based on synchronized data from the MVSA and PBS measurements, respectively. This method allowed for a comparison of the data step by step.

The data from the MVSA system was processed using a custom written Matlab^®^ script called Fodex [13]. Fodex calculates the time of HS, NHL and toe off (TO). The analysis of the PBS data was based on data from Fodex. The HS based on the PBS data was calculated as the local minima in a window of ±10% of a step length around the MVSA position of HS. The location of NHL based on the PBS data, was calculated as the maximal strain.

The measurements to validate the PBS system with the MVSA systems were performed on the test person described in Table 3. Data were collected during walking on a treadmill. The person was asked to choose his preferred walking speed [26] which was 5 km/h.

A PBS and the markers from the MVSA system were attached to the bare foot of the test person simultaneously, as shown in Figure 3(a). The markers were attached according to the procedure described in [13]. The lower part of a PBS was attached to the foot using pre-tape spray and fixation plaster. The upper part of a PBS was attached using a Velcro band.

The test person was asked to walk on a treadmill and after obtaining a consistent gait the data acquisition was started. The data were acquired over approximately 70 seconds. Retest was performed with the same test person and PBS after 2 hours. The PBS was removed from the foot and the test person was wearing a shoe between the test and retest.

### 2.4. In-Shoe Measurements Using the PBS System

The response of the PBS system to in-shoe measurements was tested using the same test person as used in the validation of the PBS system. To enable comparison of in-shoe and bare foot measurements, a bare foot measurement was acquired initially. After this recording the test person was asked to take on his socks and shoes and a new measurement was acquired. Both measurements were acquired using exact same setup and procedures as used in the validation measurements. The only factor of change was the person wearing socks and shoes during the in-shoe measurement. The test person's shoe was a white Adidas^®^ Superstar European size 42 (a common male shoe size).

The time of the characteristic events during gait (NHL and HS) on the in-shoe measurement was approximated by hand, since the MVSA system cannot be used for in-shoe measurements. The navicular drop was calculated for each step based on the events of NHL and HS on the bare foot and in-shoe measurements, respectively.

## 3. Results

### 3.1. Technical Accuracy of a PBS

**Figure 4.**
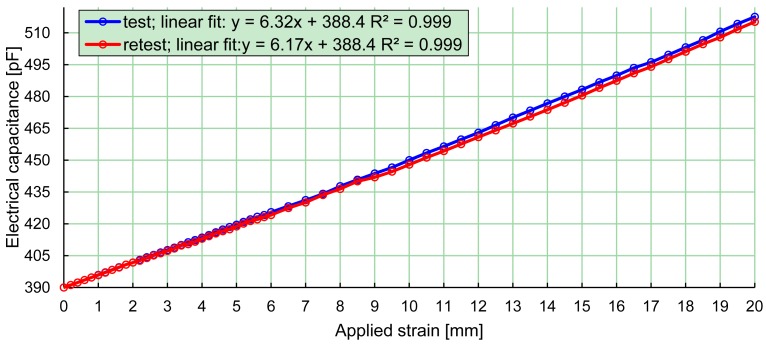
Relation between the measured electrical capacitance and strain of the PBS. Measurements were acquired using the developed system and were acquired in intervals of 0.5 mm with a strain of up to 20 mm, which correspond to a strain in the PBS of 50%.

The relationship between the electrical capacitance and strain of a PBS is illustrated in Figure 4. The PBS system was used to measure the capacitance of the PBS and the strain of the PBS was measured using a vernier caliper. The PBS was strained in maximal intervals of 0.5 mm with a strain of up to 20 mm, which corresponded to a strain of 50%. A linear fitting and the coefficient of determination was calculated for the test and the retest, respectively (Figure 4). Test-retest was performed to test the reliability of a PBS. The change in the slope of the linear fitting from test to retest was 2.5%. The test-retest is representative for a typical PBS used in the project.

**Figure 5.**
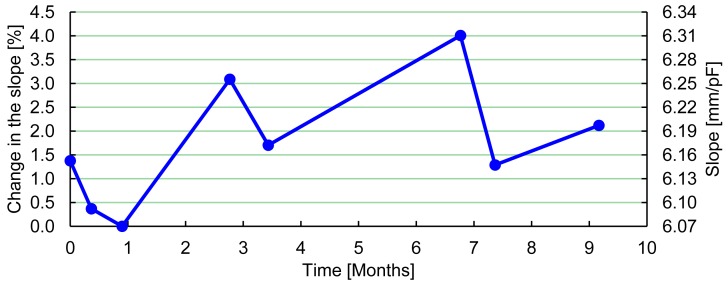
Change in the slope of a linear fitting of the relation between strain of a PBS and the electrical capacitance measured using the developed system. The graph shows the percentage change in the slope and the slope in mm/pF.

To test the change in the slope of the linear fitting over time, a typical PBS was calibrated a number of times during nine months. The capacitance was measured at two different strain values, and a linear fitting was calculated. The measurements showed a maximal change in the slope of 4% (Figure 5), which corresponds to an accuracy of 0.4 mm in a typical PBS with a navicular drop of 10 mm.

### 3.2. Validation of Navicular Drop Measurements

**Figure 6.**
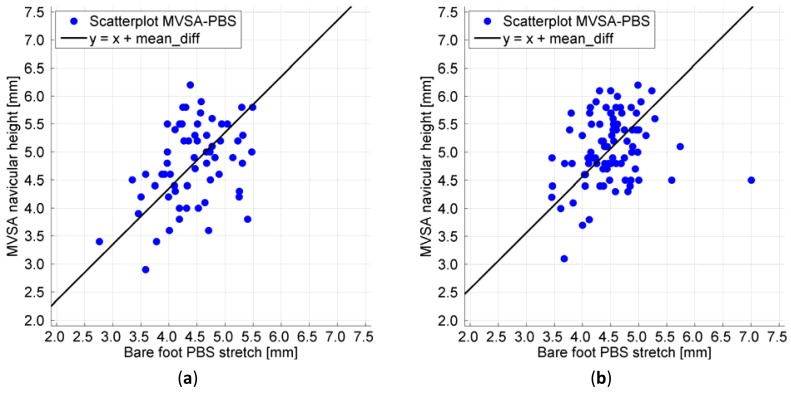
(**a**) Scatterplot test; (**b**) Scatterplot retest. The scatterplots display a similar variation around the fitted line for low and high measurements of the navicular drop, which indicates a linear relationship between PBS and MVSA measurements.

The distribution of the measurements of navicular drop measured using the PBS and the MVSA system simultaneously is illustrated in Figure 6. The black line has a slope of one and intersects the y-axis at the mean difference between the MVSA and PBS measurements of navicular drop given in Table 4. The correlation coefficient between the MVSA and PBS measurements is according to Pearsons product-moment r = 0.39 for the test and r = 0.24 for the retest. The correlations between the MVSA and PBS in the test and retest were found to be statistically significant using a significance level of 5% (*p* = 0.002 and *p* = 0.028).

The change in the mean navicular drop from test to retest using the MVSA system (Table 4) was found to be statistically significant (*p* = 0.015). The change in measurements of navicular drop from test to retest using a PBS system was not found to be statistically significant (*p* = 0.509).

**Figure 7.**
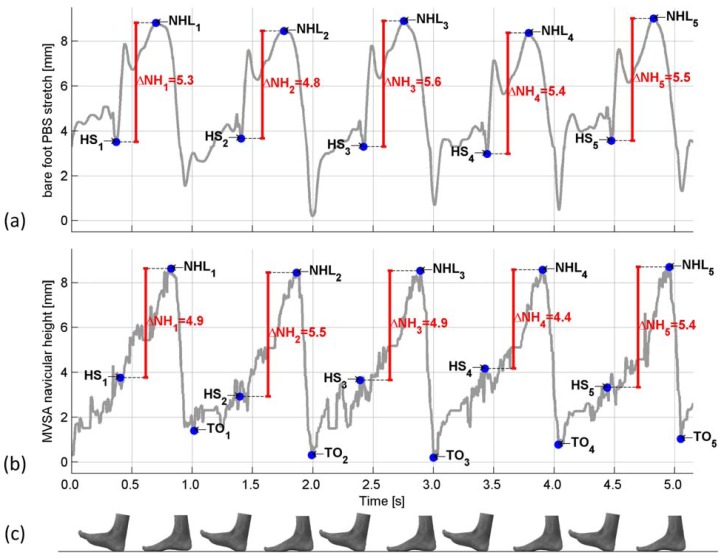
Measurements of the navicular height. The MVSA system and the PBS system are synchronized in time, using the method described in Section 2.3 (**a**) Bare foot measurement of the navicular drop using the PBS system; (**b**) Bare foot measurement of the navicular drop using the MVSA. The MVSA measurements are additive inverted, for easier comparison with the PBS measurements; (**c**) Foot indicating the stage of gait.

The navicular height measured using the MVSA system and the strain in a PBS is plotted as a function of time in Figure 7.

### 3.3. Preliminary In-Shoe Measurement of the Navicular drop Using the PBS System

**Figure 8.**
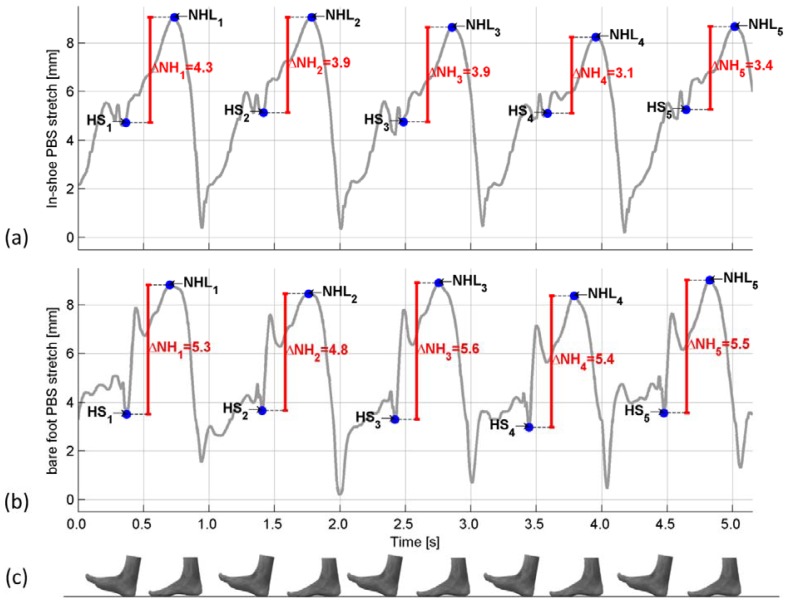
Measurements of the navicular height. The measurements were aligned by hand. (**a**) In-shoe measurement of the navicular drop using the PBS system. The sensor was found to be resistant to a person wearing a shoe. The time of the events of NHL and HS is located by hand, and for this reason the location is an estimate; (**b**) Bare foot measurement of the navicular drop using a PBS; (**c**) Foot indicating the stage of gait.

The mean In-shoe navicular drop was estimated to 3.90 mm (95%CI: 3.55;4.24) and the mean bare foot navicular drop was estimated to 5.02 mm (95%CI: 4.86;5.17). A temporal comparison of the acquired data is found in Figure 8. Calibration of the PBS was performed before and after the in-shoe measurements, and displayed no difference in technical accuracy of the sensor. No noticeable damage to the PBS was observed after the PBS was removed from the foot. While wearing the PBS the test person was asked if he noticed the PBS, and responded that he could hardly feel it on his foot.

## 4. Discussion

The purpose of the study was to develop a system that enables a dynamic measurement of the navicular drop during bare foot and in-shoe walking. The relation between the electrical capacitance and strain of a PBS was measured and the calculations of the coefficient of determination justify that the inaccuracy caused by linear fitting is negligible. Due to the linear relationship between strain and electrical capacitance, calibration of a PBS can be performed using only two calibration measurements.

The error in the calibration of the PBS observed over time was most likely caused by inaccuracy in the calibration method, where only two strain values are used to calculate the slope of the linear fitting. This calibration method was used because it reflects everyday use of the sensor, and the measurements imply that the sensor has an acceptable accuracy over time when using this calibration. To validate the PBS system with the MVSA system, measurements using the PBS system and the MVSA system were acquired simultaneously and subsequently synchronized.

The scatterplots (Figure 6) of the synchronized measurements of the navicular drop display a similar variation around the fitted line for low and high measurements of the navicular drop, which indicates a linear relationship between PBS and MVSA measurements. Pearsons product-moments show a statistical significant correlation between the measurements. Visually, some scattering is noticeable. The scattering could be caused by the slightly different movements of the foot that the PBS system and the MVSA system measures. The PBS is attached to the ankle and for this reason the movement of the ankle will have an influence on the strain of the sensor. Furthermore, a PBS is attached in a way that the sensor is in contact with the skin of the foot between the two points of attachment. This means a PBS captures a combination of both sagital and frontal plane movement of the midfoot, while the MVSA system only captures sagital deformation of the midfoot because only one camera is used. Previous research support this notion of both cyclic downwards movement and sideways movement of the navicular [28]. In addition to this we know that the movement of the navicular is a bit different from stride to stride why we would expect small changes in navicular drop during the strides [26].

The change in the mean navicular drop from test to retest using the MVSA system (Table 4) is in the range of the technical accuracy of the MVSA system, even though the change is statistically significant (*p* = 0.015). The change in measurements of navicular drop from test to retest using a PBS system was not found to be statistically significant (*p* = 0.509). This indicates that the accuracy of the PBS measurements is compatible to the accuracy the measurements from the MVSA system, which is also reflected in the better technical accuracy of a PBS.

The mean differences given in Table 4 are the differences in the mean values based on measurements from the MVSA system and PBS system, respectively. Even though the two systems capture a different movement of the foot, the difference in the mean values is in the range of the accuracy of the MVSA system, which has an accuracy of 0.5–0.8 mm [13].

The temporal comparison between the PBS and MVSA measurements (Figure 7) show that the time of HS based on the PBS measurements is very similar to the time of HS based on the MVSA measurements. The PBS based HS is characterized by a drop in the strain of the PBS. Using the fact that the NHL is the lowest height of the navicular during stance, the NHL is located very similar at the two measurements. The difference in the time of HS and NHL on the MVSA and PBS measurements could most likely be explained by the difference in the movement of the foot which the two systems capture. The difference between the navicular drop (ΔNH) measured using the MVSA system and the BPS system is in the range of the accuracy of the MVSA system [13]. Based on these preliminary measurements it is indicated that the PBS system measures the navicular drop.

We observed a decrease in navicular drop when comparing in-shoe measurements to bare-foot walking. One possible explanation could be that the contours of the insole and the shoe might support the arch of the foot and thereby decrease navicular drop [29]. This is, however, only a hypothesis and other explanations such as measurement error are also likely.

By examination of Figure 8 it is seen that the characteristic drop in strain of the PBS at HS is found in both measurements, and the maximal strain is located at approximately the same distance from HS in both measurements. Despite the preliminary character of the measurements this suggests that a PBS used in-shoe measures the same movement of the foot as a PBS used on a bare foot. Based on the validation of the PBS in the previous section this indicates that a PBS is capable of measuring the in-shoe navicular drop during walk. The calibration of the sensor before and after the in-shoe measurements and the fact that no damage to the sensor was noticed, indicates that a PBS is resistant to the influence of a person wearing a shoe.

## 5. Conclusions

In the current paper we have described the development of a strain sensor to measure navicular drop, which is a frequently used measure of foot movement.

A strain sensor based on a DEAP material from Danfoss PolyPower and a system to measure the strain of a sensor was constructed. An analysis was performed to locate an estimate of the optimal position of a strain sensor, to measure the navicular drop, and two estimated points of optimal attachment on the foot were located.

A strain sensor and markers from the MVSA system were attached to the bare foot, and measurements using both systems were acquired simultaneously to validate the strain sensor measurements. The measurements showed a linear relationship between the navicular drop measurements acquired using the MVSA and PBS system, respectively. A temporal comparison of time synchronized measurements of navicular height was also performed, and this comparison showed a similarity in the location of characteristic points of gait.

Preliminary in-shoe measurements were also acquired, and indicated that a strain sensor was resistant to the influence of a person wearing a shoe. The person wearing the sensor did not notice the sensor during walk. Temporal comparison of bare foot and in-shoe measurements indicated a similarity in the strain of the sensor. Calculations of the mean navicular drop indicated a minor decrease in navicular drop, when the person was wearing a shoe. This decrease could be explained by the shoe supporting the foot during gait.

Despite the preliminary nature of these results, the correlation between the strain of a sensor and the navicular drop has implications for future research. Clinical trials are needed to test the applicability of this device for in-shoe measurements of navicular drop in a clinical setting.

## Figures and Tables

**Table 1.** Technical data of PolyPower. Notice PolyPower is only strainable in one direction. Strain in the stiff direction will damage the material.

**Maximal strain in compliant direction:**	100%
**Maximal strain in stiff direction:**	1%
**Young's modulus:**	1.1 MPa
**Density:**	1.11 g/cm^3^

**Table 2.** Characteristics of the test person. The navicular drop given in the table is measured using the MVSA system and is a mean value based on 72 consecutive steps. The navicular drop is in the normal range of a navicular drop [12]. CI = confidence interval.

**Height**	**Foot Length**	**Weight**	**Age**	**Sex**	**Navicular Drop**
192 cm	280 mm	80 kg	25 years	male	7.2 mm (95% CI: 7.0;7.5)

**Table 3.** Characteristics of the test person.

**Height**	**Foot Length**	**Weight**	**Age**	**Sex**
175 cm	245 mm	66 kg	28 years	Male

**Table 4.** Mean values based on the measurements from the MVSA system and the PBS system, respectively. The test person has a normal navicular drop [12].

**Item**	**Test**	**Retest**
MVSA mean ND [mm]	4.8 (95%CI: 4.6;5.0)	5.1 (95%CI: 5.0;5.2)
PBS mean ND [mm]	4.4 (95%CI: 4.3;4.6)	4.5 (95%CI: 4.4;4.6)
Mean difference [mm]	0.4 (95%CI: 0.1;0.6)	0.6 (95%CI: 0.4;0.7)
Number of measurements	63	88
